# The acidosis-induced right shift of the HbO_2_ dissociation curve is maintained during erythrocyte storage

**DOI:** 10.3109/00365513.2011.565366

**Published:** 2011-04-08

**Authors:** Helge Opdahl, Tæwje A Strømme, Lise Jørgensen, Livia Bajelan, Hans E Heier

**Affiliations:** 1 Department of Acute Medicine and the Norwegian National Center for NBC Medicine, all at Oslo University Hospital, Ullevdl, Oslo, Norway; 2 Institute for Experimental Research, all at Oslo University Hospital, Ullevdl, Oslo, Norway; 3 Department of Immunology and Transfusion Medicine, all at Oslo University Hospital, Ullevdl, Oslo, Norway; 4 University of Oslo, Faculty of Medicine, Institute of Clinical Medicine, Oslo, Norway

**Keywords:** Acidosis, blood gas analysis, blood preservation, erythrocytes, haemoglobin, human, oxyhaemoglobin

## Abstract

***Background and objectives***. In fresh blood, tissue hypoxia increases microcirculatory acidosis, which enhances erythrocyte O_2_ unloading and increases the amount of available O_2_. Storage of eryfhrocytes increases the HbO_2_ affinity and reduces O_2_ unloading. We examined the development of the affinity change during a period of 5 weeks of storage by present blood bank standards, and investigated to what extent acidosis offsets the affinity change. ***Materials and methods***. Blood from volunteer donors was processed and stored as erythrocyte concentrates (EC). At 2–5 day intervals, EC were drawn from the bags and suspended in plasma and crystalloids to an Hb ≈ 10 g/dL. The suspensions were adjusted to give a pH of 7.40, 7.10, 6.80 or 6.30 and equilibrated with different gas mixtures to SO_2_ 0, 25, 50, 75 and 100%. Measurements of the PO_2_/SO_2_ pairs at each pH were used to calculate the position of the HbO_2_ curve and its P_50_ value. ***Results***. A significant leftward shift in the HbO_2_ curve was established after 1 week of storage; after 2.5 weeks only minor further changes were observed. Acidification right-shifted the HbO_2_ curve, after 2.5 weeks of storage the curve at pH 7.10 was similar to that for fresh blood at pH 7.40. Calculations of extractable O_2_ showed that the left-shifted HbO_2_ curve of stored EC could be advantageous at a low arterial PO_2_. ***Conclusions***. The rightward shift of the HbO_2_ curve due to acidosis is well maintained in stored eryfhrocytes, a moderate pH decrease offsets the storage-induced increased HbO_2_ affinity.

## Introduction

The O_2_ content of arterial blood (CaO_2_) is mainly determined by the amount of haemoglobin (Hb) and its O_2_ saturation (SaO_2_); the number of O_2_ molecules dissolved as gas and measured as the O_2_ partial pressure (PaO_2_) represents under normal circumstances only 1-2% of the total. The importance of PaO_2_ for O_2_ delivery to the tissues lies therefore not in the PaO_2_ *per se*, but in its effect on the SaO_2_. The relationship between PO_2_ and SO_2_ is described by the sigmoid-shaped HbO_2_ dissociation curve; at a constant PO_2_ a leftward shift in the curve position increases the SO_2_ and thus the CaO_2_ while a right-ward shift decreases the SO_2_. Due to the shape of the HbO_2_ curve, the effects of such shifts are usually miniscule at normal or high PO_2_ levels but may be crucial at low PO_2_ levels.

During blood bank storage of erythrocytes, depletion of their 2,3 diphosphoglycerate (2,3-DPG) content [1] shifts the HbO_2_ curve to the left.This increases the CaO_2_, but will also decrease the unloading of O_2_ in the tissue microcirculation. Some authors have considered this a major argument against a beneficial effect of transfusion of stored erythrocytes [2,3], and others have suggested that only fresh erythrocytes should be transfused to intensive care patients [4]. However, others have pointed out that experimental and clinical studies have yielded controversial results on the eventually negative effect of transfusing stored erythrocytes, and that many more factors than the actual position of the HbO_2_ dissociation curve influence the clinical effect of a red cell transfusion [5].

In fresh blood, tissue acidosis will increase O_2_ unloading; hypoxic acidosis will thus automatically increase tissue O_2_ delivery. To what extent acidosis can compensate for the storage-induced leftward shift in erythrocyte concentrates (EC) preserved in SAGMAN solution has not previously been determined. In the massively transfused patient, the circulating blood consists of a mixture of transfused EC and plasma from the blood bank plus infused electrolyte solutions. The O_2_ transport capability and tissue unloading of such blood during the acute phase is therefore equivalent to that of stored EC.

Calculations of curve shifts based on standard pH-dependent correction factors do not correct for changes in 2,3-DPG; even in fresh blood such calculations become increasingly inaccurate with mounting acidosis [6]. We therefore measured the storage-induced effect on the HbO_2_ curve shifts in EC at 2–5 day intervals of storage for up to 35 days; on each experimental day, the samples were examined at four different pH values. To simulate *in vivo* conditions during major bleeding, the stored erythrocytes were resuspended in equal amounts of plasma and crystalloids to an Hb of 9–11 g/dL. The consequences of the storage dependent HbO_2_ curve shifts for the amount of consumable O_2_ [7] during threatening tissue hypoxia were also calculated.

## Materials and methods

### Sources of blood

Eighteen healthy persons from the donor pool of Oslo University Hospital Blood Bank (OBB) received oral and written information about the investigation and gave written consent to allow their donation on a particular day to be used for research purposes. During preliminary methodological studies, blood from one of the authors (HEH) and samples from outdated blood to be discarded were also used. The investigation was approved by the Regional Committee for Medical research ethics, the Biobank committee and the Committee for Individual rights in research.

The blood to be used in the investigation was collected, processed and stored at 4°C by procedures identical to those routinely employed for preparation of erythrocyte concentrates (EC) at the blood bank. Briefly, 450 mL whole blood was mixed with 63 mL of citrate-phosphate-dextrose-adenine (CPD-A) in a plastic bag (Fenwal Corp., Illinois, USA) and left at room temperature for 1 h. It was then centrifuged at 4°C and 4000 *g* for 20 min, followed by removal of plasma and buffy coat into separate bags by pressure. The remaining erythrocyte concentrate was passed through a leukocyte filter (Asahi Kasei Medical Co, Japan) at room temperature, resuspended in SAGMAN solution (Fenwal) and immediately stored at 4°C. The procedure until storage typically took 4–5 h and never exceeded 8h. EC produced at the OBB show a mean volume = 245 mL, a mean haematocrit =0.55 and a mean haemoglobin content = 49 g/bag (unpublished quality control data).

**Figure 1:**
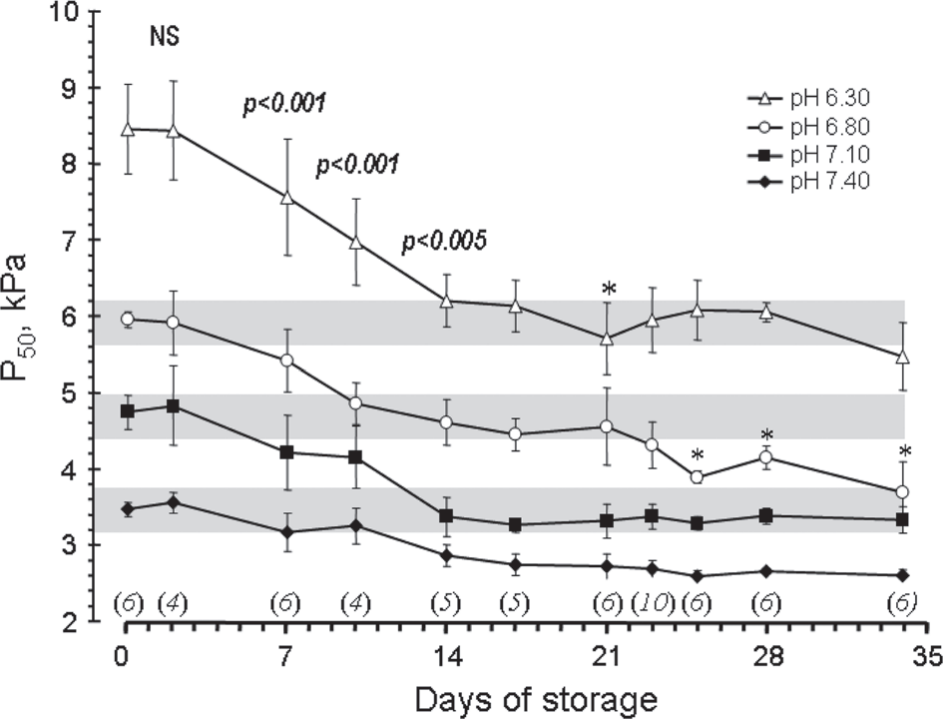
Changes in P_50_ values as a function of storage time measured at the four different pH values shown in the figure. All data from 0–17 days of storage represent blood from the same EC bags. The number of EC bags examined after each storage period is shown in italics above the x-axis. Data for all storage periods longer than 2 days were significantly different from day 0. The *p* values shown as numbers in the figure refer to a difference from the preceding P_50_ level by a paired T-test including all pH levels. The asterisks denote a significant (*p < 0.05) difference from the preceding value when each individual pH level was analysed separately.

The HbO_2_ affinity of EC stored at 4°C was examined after different time intervals. On day 0, samples of EC processed for storage the same day were examined before cooling and storage. During the next 3 weeks of storage, samples of EC from the same six donors were examined at time intervals as shown in Figure 1. The necessary volume of EC from each donor bag was transferred aseptically into a smaller bag (‘Baby bag’, Fenwal) on each experimental day, after which both bags were shut by welding. Due to the limited volume of the EC bags, the contents of each bag could be followed only for up to 3 weeks. Therefore, blood from additional donors was necessary to obtain data corresponding to a storage time of 3-5 weeks.

In 6 donors, an additional 15 mL of blood was drawn separately into heparinized tubes for estimation of both the normal HbO_2_ curve and that representing grave acidosis at approximately pH 6.30 in non-processed blood. The position of the HbO_2_ curve at pH 6.30 calculated from these data was in agreement with that previously determined in a detailed investigation in our hospital [6] and served to verify the method for HbO_2_ curve determination used in the present investigation. The choice of anticoagulant or the processing of blood to EC had no significant effect on the HbO_2_ curve within the normal range of pH values. All laboratory work, including tonometry and blood gas analysis, was carried out in the facilities of the blood bank.

### Preparation of blood samples

Five mL of EC was mixed with 3 mL of thawed Octaplas® (pooled, standardized, solvent/detergent virus inactivated whole plasma) of blood group AB, the pH, base excess (BE) and other blood gas parameters of this mixture were then measured in an ABL 700 blood gas apparatus (Radiometer Medical, Bronshoj, Denmark) equipped with a co-oximeter. Subsequently, 3 mL of an electrolyte mixture was added, giving an Hb of 9–11 g/dL in the final solution. The mixture consisted of sterile H_2_O (Fresenius Kabi, Halden, Norway) and lactic acid (Sigma Aldrich, St Louis, MO, USA) *or* NaHCO_3_ 0.5 mol/L (Braun Melsungen, Melsungen, Germany). The amount of lactic acid or NaHCO_3_ was calculated from the initial pH and BE values to give pH levels of approximately 7.40, 7.10, 6.80 or 6.30 in the final EC-PE mixture. In addition, concentrated NaCl (4 mol/L, Addex NaCl, Fresenius Kabi, Halden, Norway) was added in amounts calculated to give a final volume of 3 mL and a Na^+^ of 140 mmol/L in the electrolyte mixture. The accuracy of the calculations was verified by the electrolyte analysis in the final EC-plasma-electrolyte (EC-PE) mixture given by the blood gas apparatus. In experiments involving heparinized blood no plasma was added; the effect of acidification to pH 6.30 was examined after adjusting the pH by addition of 3 mL of electrolyte mixture by a procedure identical to that described above.

Both processing and storage acidify the EC. Preliminary experiments showed that additions of acid or NaHCO_3_ aimed to give pH values of 7.40 and 7.10 could be calculated from the Siggaard-Andersen acid-base nomogram [8] with reasonable accuracy. The nomogram proved inaccurate, however, when the goal was pH 6.80, and a pH of 6.30 was out of its range. Separate preliminary experiments were therefore necessary to establish empirical addition factors. Obtaining the exact designated pH value in the lower pH range proved difficult and time consuming; the median pH value deviation from the designated value for each group was, however, always less than 0.1 pH units. For simplicity, the designated pH values are shown in figures and text.

### Tonometry and measurements

The EC-PE mixture was divided into two 5 mL plastic syringes (3.5 mL each) and placed in a tonometer (RNA medical equilibrator model 300, RNA Medical, Massachusetts, USA) preheated to 37° C. The syringes, which were pretreated with a foam-inhibiting coating by the tonometer manufacturer, were equilibrated with a gas containing 5% CO_2_ and 20% O_2_ in nitrogen *or* 5% CO_2_ in nitrogen, respectively, for 20 min. The EC-PE mixture from the two syringes had an SO_2_ of 90–100% (depending on the pH) or close to 0%, respectively, with a pCO_2_ of approximately 5 kPa. The contents of the two syringes were then transferred anaerobically into 2 mL syringes containing a glass bead for mixing purposes, in amounts calculated to give the final solutions an SO_2_ of approximately 25%, 50% and 75%, respectively. The final mixtures, as well as the remaining ≈0% and ≈100% blood, were then analysed in the blood gas apparatus. The five sets of corresponding SO_2_–PO_2_ values at each pH were then used for determination of the position of the HbO_2_ curve. The PO_2_ corresponding to half saturation of the Hb with O_2_, the P_50_ value, were calculated from our data (see below) and used to express changes in the HbO_2_ curve position.

### Estimation of storage and pH effects on the HbO_2_ curve and P_50_ values

Often used equations (Siggaard-Andersen, Severing-haus) for calculating changes in the HbO_2_ curve induced by variations in pH overestimate the right-ward shift of the HbO_2_ curve in grave acidosis [6]. A dedicated data program using LabView software was developed locally by one of the authors, a research data engineer (TAS). The program fitted a sigmoid shaped curve to the position of the five PO_2_–SO_2_ measurement pairs at each pH; this curve was then compared to a population of HbO_2_ curves calculated by the equations proposed by Severinghaus [9] and by Kellman [10] for determination of the effect of pH changes on the HbO_2_ curve position. The curve representing the best fit to the measured data was chosen as the ‘true’ HbO_2_ curve. Curves calculated according to the Kellman method gave the best visual fit to the observed PO_2_–SO_2_ data, the pH of which the curves were calculated also deviated least from the actual pH of the samples. This routine was therefore utilized for determination of the position of the curve as defined by its P_50_ value. The median P_50_ value for heparinized blood at a pH of 6.30 calculated by this method (8.9 kPa) was very close to that previously determined in our institution (9.1 kPa) by multiple direct measurements in heparinized blood at the same pH [6], thus verifying our method for determining the position of the HbO_2_ curve even in grave acidosis.

### Calculation of consumable oxygen

At an end venous PO_2_ below 2.7 kPa (=20 mmHg, see Conversions section below), cells may become dysfunctional and lactic acid production can be induced [6,11]. As more than 98% of the O_2_ in normal arterial blood is bound to Hb, the amount of consumable O_2_ calculated for a particular HbO_2_ dissociation curve can, for simplicity, be expressed as the difference between the SO_2_ corresponding to that of a given arterial PO_2_ level and that at a PO_2_ of 2.7 kPa. For the purpose of such calculations, PaO_2_ levels corresponding to grave hypoxemia (5.3 kPa, 40 mm Hg), normoxia (13.3 kPa, 100 mm Hg) and supernormal O_2_ levels (20 kPa, 150 mmHg) were used. These calculations assume a similar pH in arterial and capillary blood. Under extreme conditions, however, major differences between arterial and microcirculatory pH levels may exist (see discussion). Therefore, calculations of consumable O_2_, assuming a HbO_2_ curve position corresponding to pH 7.40 in arterial blood and to pH 6.80 locally in the microcirculation of hypoxic tissue, were also carried out.

### Conversions

To facilitate comparisons of P_50_ values between our results and those of other authors, data originally given in mmHg or Torr is converted to kPa by the factor 1 kPa = 7.5 mmHg or Torr.

### Statistics

Data representing P_50_ values (Figure 1) are shown as group medians with their standard deviation. For statistical purposes, data from adjacent days of storage were pooled, data shown in Figure 1 as days 7, 14, 17, 21, 23, 25, 28 and 34 represents days 7–8, 13–14, 16–17, 20–21, 22–23, 24–26, 27–28 and 32–35 days, respectively. The number of stored EC bags utilized within each storage period is shown in Figure 1.

As analysis of raw data showed them to be normally distributed, parametric methods were utilized. A one-way ANOVA analysis was initially carried out for all storage times and each pH level. As all data from the first 16–17 days were obtained from samples originating from the same EC bags, a paired T-test was used for comparison of P_50_ values for the different storage periods during this period. Independent samples T-test was utilized for comparison of the longer storage periods, where the variations in donors on different storage days were greater. Regression analysis was used to analyse the relation between the pH and P_50_ values at each week of storage, differences in regression lines for the pH-P_50_ plots were based on differences between the 95% confidence intervals for each line. All analyses were carried out by means of the SPSS statistical software package, version 15.

## Results

### Changes in HbO_2_ dissociation curve during storage

**Figure 2:**
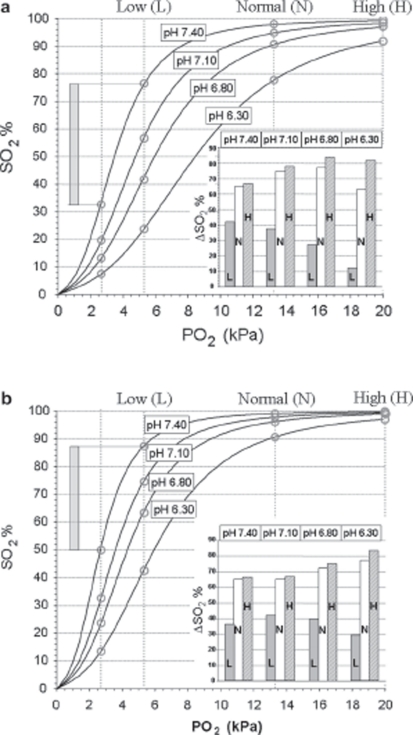
Actual HbO_2_ dissociation curves at 4 different pH values in blood from EC before storage (a) and after storage for 16–17 days (b). Vertical lines are drawn corresponding to PO_2_ values of 2.7 kPa (critical end-venous level), 5.3 kPa – Low (L), 13.3 kPa – Normal (N) and 20 kPa – High (H). The intersection between these lines and the HbO_2_ curves are marked with circles, the amount of consumable oxygen given as the ΔSO_2_ values depicted in the inserts was calculated as exemplified by the method shown for pH = 7.40 and PO_2_ = 5.3 kPa in both figures.

The median P_50_ in the EC-plasma-electrolyte mixture on the day of blood donation was 3.47 kPa at pH 7.40, 4.75 kPa at 7.10, 5.96 kPa at pH 6.80 and 8.45 kPa at pH 6.30 (Figure 1). The actual HbO_2_ dissociation curves corresponding to the P_50_ values are depicted in Figure 2a. There was no detectable change in P_50_ values during the first 2 days of storage; from 7 days and onwards, the P_50_ decreased (i.e. HbO_2_ affinity increased) for all pH levels (*p* < 0.001 for all pH levels pooled, *p* < 0.05 for analysis of each pH level separately). Analysis of changes in P_50_ values from the previous observation during storage showed a significant (see Figure 1) further decrease during the second week of storage. After 16–17 days, however, the P_50_ at all pH values was stabilized (see Figure 2b for actual HbO_2_ curves on days 16–17 of storage). The minor changes for the pH 6.80 level at the end of the storage period could not be detected for the other pH levels, and probably represent only random effects.

The magnitude of the P_50_ change, defined as the difference between the P_50_ values at day 0 and at day 16–17, was more marked in acidotic blood than at normal pH (Figure 1), as could be expected from graphic presentations of pH-dependent curve shifts. The HbO_2_ leftward shift measured at pH ≈7.40 corresponded to a P_50_ change of approximately 0.68 kPa, with a curve position equal to that of fresh blood at a pH of ≈7.72 (Figure 2b).The leftward shift of P_50_ at pH ≈7.10 was 1.50 kPa, corresponding to normal blood at pH ≈7.40. At pH ≈6.80 the P_50_ shift was 1.45 kPa, corresponding to normal blood at pH ≈ 7.20. There was no statistical difference between the shifts at pH 7.10 and 6.80, but both were larger than at pH 7.40 (*p* < 0.001). At pH ≈6.30 the P_50_ shift was approximately 2.3 kPa, which was greater than that for the other pH values examined (*p* < 0.001 compared to pH 7.40, *p* < 0.05 compared to pH 7.10 and 6.80). The shift at this pH gave a P_50_ value corresponding to that of fresh blood at pH ≈6.85.

### Consequences of erythrocyte storage for consumable oxygen

The amount of consumable O_2_, expressed as the difference in SO_2_ between that corresponding to the PO_2_ of arterial blood and a microcirculatory end-venous PO_2_ of 2.7 kPa (ΔSO_2_), was calculated from the HbO_2_ dissociation curves on storage day 0 (Figure 2a and insert) and after the affinity change had stabilized at 16–17 days (Figure 2b and insert). On day 0, the amounts of consumable O_2_ at high (20.0 kPa, 150 mm Hg) and normal (13.3 kPa, 100 mm Hg) PO_2_ levels increased with mounting acidosis from pH 7.40 to pH 6.80, but started to decline when pH fell to 6.30. At low PO_2_ (5.3 kPa, 40 mm Hg), corresponding to grave clinical hypoxemia, the consumable O_2_ decreased continuously with increasing acidosis. After storage for 16–17 days, the amount of consumable O_2_ at high and normal PO_2_ followed the same pattern as on day 0, but was reduced by almost 20% at pH 7.40 and about 10% at pH 7.10. At extreme acidosis of pH 6.30, however, the amount of consumable O_2_ was higher after storage, by about 2% at high and by 14% at normal PaO_2_. At the low PO_2_ level, the amount of consumable O_2_ at pH 7.40 was slightly smaller after storage than on day 0; the increased HbO_2_ affinity of storage proved, however, progressively beneficial compared to day 0 with mounting acidosis (ΔSO_2_ at pH 7.10: 42.0% *vs* 37.5%, at pH 6.80: 39.9% *vs* 27.3% and at pH 6.30: 29.1% *vs* 12.1%, respectively).

**Figure 3:**
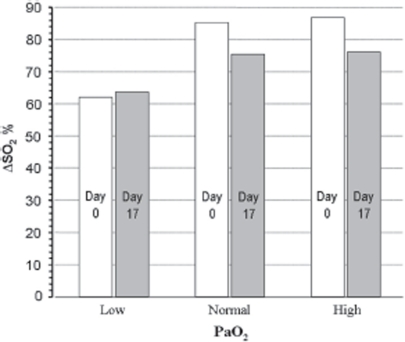
Consumable oxygen, assuming a pH 7.40 in arterial blood and pH 6.80 in the microcirculation, calculated for Low, Normal and High PO_2_ values as in the inserts in Figure 2a and b.

Similar calculations, but assuming that the arterial blood was maintained at pH 7.40 while that in poorly oxygenated tissue was acidified to pH 6.80 (see methods), are shown in Figure 3. Again, storage reduces consumable O_2_ at high and normal PaO_2_ values, but to a lower degree than that calculated for a constant pH. Storage will have a slight beneficial effect at very low PO_2_ (ΔSO_2_ 63.6 % *vs.* 62.1 %).

### P_50_ as a function of pH

**Figure 4:**
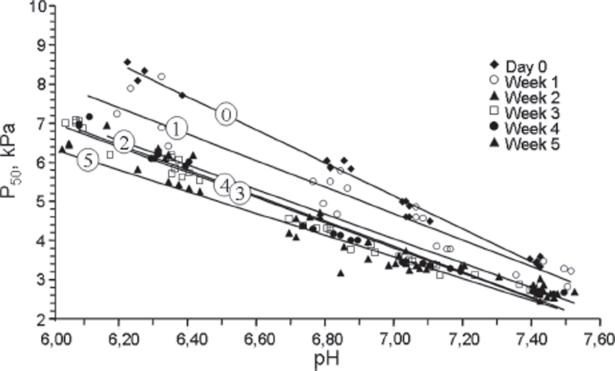
The relationship between pH and P_50_ values at 0, 1, 2, 3, 4 and 5 weeks of storage shown as a scatterplot, symbols as shown in the figure. The trend lines for each period are numbered corresponding to weeks of storage.

The relationship between pH and P_50_ was almost linear within the acidotic pH range compatible with survival, both initially and after storage (Figure 4). Analysis of regression with pH as the independent variable showed a strong correlation before storage (r^2^ = 0.981, *p* < 0.001), as well as after 1 week, (r^2^ = 0.928, *p* < 0.001), 2 weeks (r^2^ = 0.974, *p* < 0.001), 3 weeks (r^2^ = 0.981, *p* < 0.001), 4 weeks (r^2^ = 0.978, *p* < 0.001) and 5 weeks (r^2^ = 0.961, *p* < 0.001).The rise rate was steeper on day 0 before storage, with essentially similar slopes at 1 week of storage and for longer storage periods.The 95% confidence interval for the pH-P_50_ regression line on day 0 was different from those for 2 weeks and onward (−4.146, −3.681 vs. −3.384, −2.887 at 2 weeks, −3.228, −2.788 at 3 weeks, −3.458, −3.060 at 4 weeks and −3.006, −2.546 at 5 weeks of storage). The difference between the lines at day 0 and 1 week of storage (−3.774, −2.946) was borderline.

## Discussion

The P_50_ value in fresh blood at pH 7.40 is usually given as 3.47–3.87 kPa (26–29 mmHg) [12,13]. In our study, the P_50_ in the EC suspension after the processing procedure (anticoagulation and separation) for EC employed in a modern blood bank corresponded to the lower value, and did not change after cooling and storage for 2 days. A leftward shift of the HbO_2_ curve became evident after 7 days of storage and became stabilized after 2.5 weeks. A storage-induced leftward shift of the HbO_2_ curve in whole blood was first quantified by Valtis and Kennedy in 1954 [14] and later confirmed by others [15,16]. In blood processed and stored by the methods common at that time (ACD blood), the P_50_ after 15–20 days of storage, when calculated back to conditions at pH 7.40, was found to be lower than that of EC in our investigation. The magnitude of these differences are moderate, from 0.3 kPa (2 mmHg) [14] to 0.7 kPa (5 mmHg) [16]. In contrast to our data, two of the previous studies [14,15] found that most of the change in the HbO_2_ curve occurred within the first 7 days of storage, while another [16], analogous to our data, found that the bulk of the P_50_ change occurred within the first 15 days. The differences in the magnitude of change and the time pattern may, at least in part, be attributed to the different routines for erythrocyte preservation in use during the time when the investigations were carried out.

The main cause of the affinity change has been identified as a reduction in erythrocyte 2, 3-diphosphoglycerate (2,3-DPG) [1] during blood bank storage. A previous investigation in our blood bank, utilizing blood processed and stored by the same routine as in our experiments, showed that the erythrocyte 2,3-DPG levels were well maintained during the first 24 h, but declined to 60–80% during the first week of storage and further reduced to 30–40% of the initial value after 2 weeks of storage. A further reduction to about 20% was seen after 25 days [17]. As the leftward shift of the HbO_2_ curve seems to have stabilised after 2–2.5 weeks in our study, the last 20% reduction of 2,3-DPG seems to have little impact on the P_50_ of the stored blood. Incubation of erythrocytes with phosphate mixtures may increase the erythrocyte content of 2,3 DPG. In a clinical setting involving urgent need for massive transfusions, such pre-treatment of stored erythrocytes would be impractical.

Some authors have considered the reduced O_2_ unloading ability of blood with a leftward curve shift as a major argument against a beneficial effect of bank blood transfusions [2,3]. In our study, adding H^+^ to the erythrocyte solution could abolish the HbO_2_ affinity change induced by blood bank storage. In clinical settings involving massive transfusions (e.g. major trauma), arterial and/or tissue acidosis can be expected; the transfused blood would then have an O_2_ unloading capability similar to that of a person's own blood at normal pH.

Whether an HbO_2_ curve shift has negative or positive consequences for tissue O_2_ delivery depends on the mechanisms underlying threatening or manifest hypoxia in each particular patient. A rightward shift of the HbO_2_ curve increases O_2_ unloading in the microdrculation; at extreme, but clinically relevant acidosis [18], the microcirculatory SO_2_ may fall to 7–8% before the PO_2_ decreases below 2.7 kPa (20 mmHg) [6]. This makes almost all O_2_ in the blood available to the tissue cells. On the other hand, it decreases the SO_2_ corresponding to a given PO_2_ in the blood leaving the lungs. This effect is of minor significance if the acidosis is moderate and the PaO_2_ levels are in the normal or supranormal range. At an extreme pH of 6.30, however, a normal PaO_2_ of 13.3 kPa (100 mmHg) results in an SaO_2_ of only 76–77%, and a PaO_2_ of approximately 30 kPa (225 mmHg) is necessary to obtain a normal SaO_2_ [6]. The effect of acidosis-induced rightward shift on SaO_2_ is accentuated if the patient also has respiratory insufficiency. A PaO_2_ of 7.0 kPa (52.5 mmHg), which at normal pH would give a SaO_2_ of 87 %, resulted in a SaO_2_ of 45% in a patient with a pH of 6.66 [19].

Under normal conditions, the CO_2_ released by tissue cells induces a slight acidification and thus a minor rightward shift of the HbO_2_ curve as arterial blood enters the microcirculation (the ‘Bohr shift’). The shift is reversed when CO_2_ is excreted by the lungs. During episodes of threatening or manifest tissue hypoxia, the optimal condition for transport and utilization of O_2_ would be a left-shifted HbO_2_ curve position in the blood during passage through the lungs (ensuring optimal CaO_2_), and a major shift to the right in the microcirculation (ensuring optimal tissue PO_2_). During circulatory failure or maximal exercise, a local tissue H^+^ concentration up to ten times higher than that in normal arterial blood may develop [20,21]. If the amount of the locally generated excess acid metabolites is within the elimination capability of the organism, this acidosis will not necessarily be reflected in arterial blood [21,22]. As illustrated by Figure 3, a situation in which arterial pH is 7.40 and that in the most vulnerable tissues is 6.80, the amount of consumable O_2_ at the very low PaO_2_ of 5.3 kPa may be maintained at a level close to that calculated for a normal PaO_2_ at a pH of 7.40.

Under conditions where the PaO_2_ is very low, a leftward shift of the HbO_2_ curve (see Figures 2a and b) may be necessary for survival. During vaginal delivery the baby's PaO_2_ may decrease to values in the 2.9–3.33 kPa (22–25 mmHg) range [23], a leftward HbO_2_ curve shift due to fetal Hb makes a SaO_2_ in the 60–70% range possible. A marked leftward shift, induced by arterial blood alkalosis, is also a prerequisite for maintaining an acceptable SaO_2_ during high altitude ascents without supplementary O_2_ [24,25]. Theoretically, a left shifted HbO_2_ curve could therefore also be advantageous in catastrophic lung failure with very low PaO_2_ values.

The increased affinity for O_2_ in erythrocytes stored for up to 35 days in our study was reversed by acidification of the suspension medium. The range of pH values chosen for examination in our study is clinically relevant. Most clinicians will treat a metabolic acidosis before it reaches pH 7.10 [26–28], especially in unstable patients, a pH of 6.80 is often cited as the lower limit for expected survival [29,30]. Nevertheless, many clinicians have successfully treated patients with even lower values; ultimate survival and restitution with a pH value of 6.33 in arterial blood has been reported [18]. Equal, or even lower pH values may exist locally in hypoxic tissue [20,21]. In our study, acidification right-shifted the HbO_2_ curve of stored EC close to that of fresh blood; after 2.5 weeks of storage the curve at pH 7.10 was similar to that of fresh blood at pH 7.40.

The term consumable (or extractable) O_2_ has been used to denote the amount of oxygen that can be extracted from the blood before tissue cells becomes dysfunctional because of hypoxia [31,32], calculations of this parameter give an indication of the consequences of HbO_2_ curve shifts for O_2_ tissue supply. The results of such calculations are not exact, as the use of a lower borderline value of 2.7 kPa (20 mmHg) is somewhat arbitrary and direct measurements of tissue PO_2_ levels vary considerably [33–35]. Our calculations indicate, however, that the left-shifted HbO_2_ curve of stored EC could be advantageous at a low arterial PO_2_. Since the pH-dependent change in Hb affinity for O_2_ is largely conserved in stored blood, the leftward shift of the HbO_2_ curve in stored EC probably has no deleterious effect in most patients.

## Conclusion

As the leftward HbO_2_ curve shift occurring during storage does not abolish the rightward shift induced by acidosis, the increased HbO_2_ affinity of EC stored for 2 weeks or more does not represent a major tissue oxygenation problem in most transfused patients.
